# Trends in the Endocrinology Fellowship Recruitment: Reasons for Concern and Possible Interventions

**DOI:** 10.1210/clinem/dgaa134

**Published:** 2020-03-19

**Authors:** Giulio R Romeo, Irl B Hirsch, Robert W Lash, Robert A Gabbay

**Affiliations:** 1 Joslin Diabetes Center, Harvard Medical School, Boston, Massachusetts; 2 Division of Endocrinology, Diabetes and Nutrition, University of Washington, Seattle, Washington; 3 Endocrine Society, Washington, District of Columbia

**Keywords:** fellowship recruitment, physician-scientist, endocrinology workforce, job satisfaction, loan forgiveness

Trends in US physician workforce supply and demand have been analyzed since the 1960s using a combination of expert panel opinions, modeling of historical and original data, and physician surveys.

While establishing the optimal trajectory for the “stock” of physicians is inherently difficult, projections of a surplus of specialists emerging from some early reports (1,2) have not held true for many internal medicine (IM) subspecialties.

Indeed, the current shortage of endocrinologists was already predicted in 2003 by findings of the Lewin Group, which had been commissioned by the Endocrine Society to determine the endocrinology workforce needs over the next 2 decades (3). Never more so than today, the forecasted widening gap of demand–supply between 2010 and 2020 proved both accurate and alarming. A more recent analysis projects that, in the absence of any intervention, the shortage of adult endocrinologists will increase to ~2700 by 2025 (4).

In addition to attrition (primarily retirement rate), the number of filled endocrinology fellowship positions (the “pipeline”) is the main factor influencing the prospective workforce. The ~50% expansion in endocrinology training positions from 223 in 2009 to 326 in 2019 (5) has been accompanied by a stagnant number of applications, as underscored by the decline in the applicants/positions ratio that is currently approaching 1.0 (Fig. 1). Of concern, this sustained downturn is materializing at a moment of conspicuous escalation in the demand for endocrinologists.

**Figure 1.**
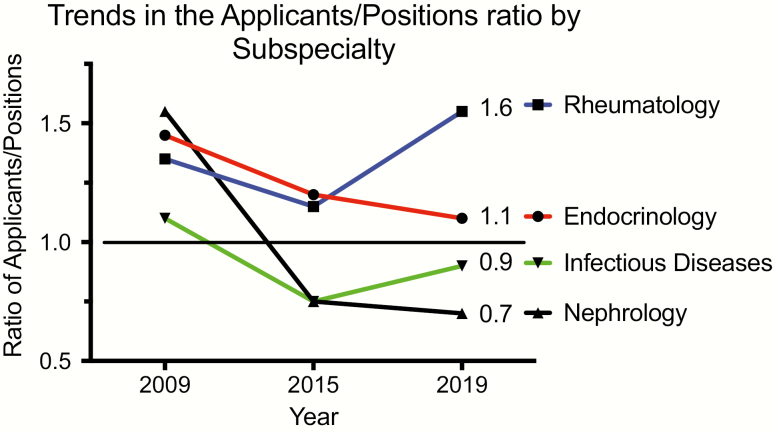
Trends in the ratio of applicants/positions for 4 IM subspecialties. With different patterns, Endocrinology, Nephrology, and Infectious Diseases have all experienced reduced competitiveness since 2009, whereas Rheumatology has recently become more competitive.

Similar to other IM subspecialties, significant changes have occurred in the composition of recent classes of endocrinology fellows that herald major shifts in the future workforce. First, the proportion of female fellows has risen over the past 15 years, constituting 71% of the entering cohort in 2016 (6), in the context of a steady reduction of male enrollees (7). As discussed below, this trend should force the medical community at large to address the gender pay gap and biases in career advancement as a means to ensure retention of the whole workforce. Second, data from the American Board of Internal Medicine reveal a switch from US Medical Graduates (USMGs) to International Medical Graduates (IMGs) as the predominant component of first-year endocrinology fellow classes since 2013, with IMGs accounting for ~60% in 2019 (8) (Fig. 2). Albeit long-term implications of such change are unclear, potential visa or work permit restrictions may affect job opportunities for many graduating endocrinologists. Third, the percentage of endocrinology fellows pursuing a 3-year, research-oriented training versus a 2-year program continues to plummet: data extracted from the National Resident Matching Program show a decline from ~23% in 2015 (59/252 positions filled) to 15% in 2019 (46/306), which will further affect the thinning pool of physician-scientists (9). Multiple factors contribute to this trend, including a more competitive funding environment, perceived uncertainty of career trajectory as an investigator, and, for many IMGs, eligibility limitations for research support during fellowship (eg, T32) that de facto prevent them from entering a 3-year track.

**Figure 2.**
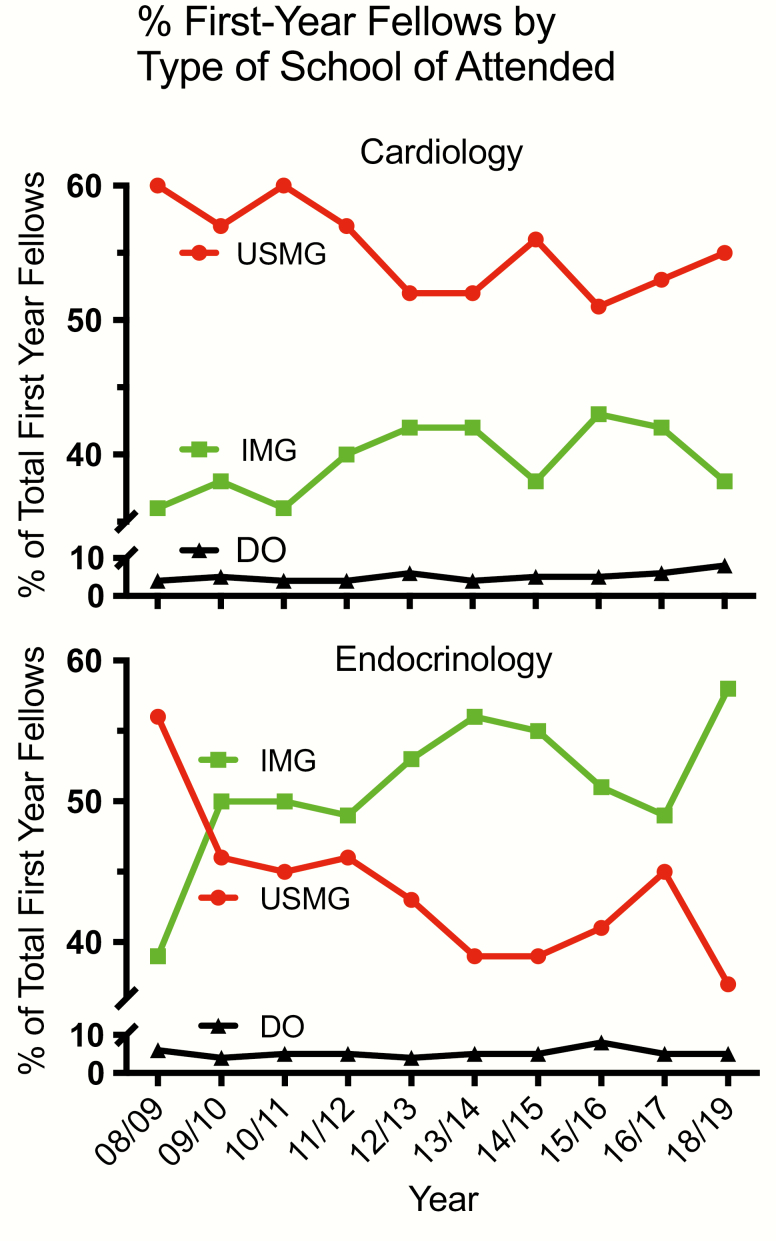
Trends in the percentage of first-year fellows over time for Endocrinology (bottom) and Cardiology (top) as US Medical Graduates (USMGs), International Medical Graduate(IMGs), and Doctor of Osteopathic medicine (DO). Since 2013 IMG have contributed the majority of first-year endocrinology fellows whereas USMG consistently represented >50% of incoming cohorts of cardiology fellows. The percentage accounted for by the DO group has remained stable at <10% for both specialties.

Rather than a comprehensive review of supply–demand dynamics, here we plan to (1) address root causes for the waning interest in entering a career in Adult Endocrinology, (2) discuss consequences of the reduced workforce supply, and (3) propose interventions aimed at halting and inverting this trend.

## Push and Pull Factors Affecting the “Pipeline”

**Figure 3.**
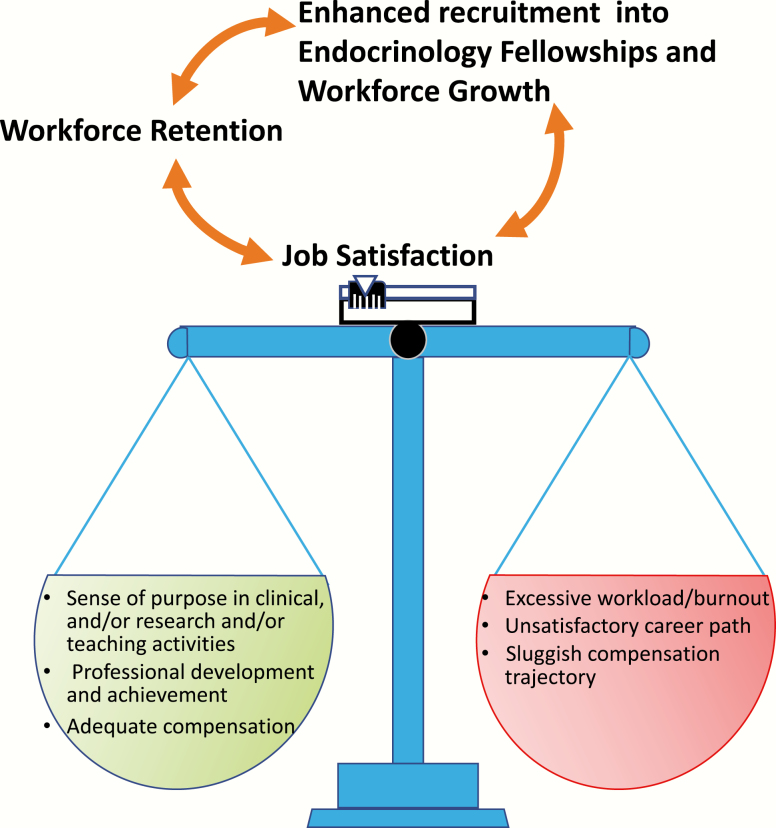
Job satisfaction is represented as an integrated measure of multiple factors, each with different “weight,” which determine the individual’s balance. In this model, job satisfaction is a key aspect for workforce retention and for increasing the appeal of endocrinology fellowship training.

What key reason(s) will entice or dissuade medical students (MSs) and IM residents through their training to choose a career in Adult Endocrinology? In a nutshell, the perceived likelihood of job satisfaction, as a measure of the balance of workload, lifestyle, compensation, professional development, and relationship with patients (Fig. 3).

On the one hand, intellectual stimulation and value of longitudinal relationship with patients are core factors attracting budding endocrinologists (10). Other relevant aspects include control of lifestyle (11), and practice characteristics (eg, largely “nonprocedure” based) that align with personality traits (12). On the other hand, the lowest compensation among subspecialties (13), increasing time spent on paperwork and the exponential pressure to see more patients are major disincentives.

To dissect why endocrinology has become such an unpopular career choice, especially among US graduates, certain factors deserve particular attention.

### Early exposure to endocrinology

As part of a vicious cycle, the rise of administrative tasks and clinical responsibilities restrict the time dedicated to teaching, which is essential to attract and nurture the next generation of endocrinologists. This issue is often compounded by scarce exposure to endocrinology through medical training. For MSs, the introduction to endocrinology is routinely limited to inpatient glucose control, which is often relegated to one of the low-priority items on rounds. The evaluation and treatment of the more interesting endocrine disorders and longitudinal diabetes care is typically done in the outpatient setting, where it is unusual to find a third- or fourth-year MSs.

The challenges are only amplified during IM residency. Inpatient endocrine rotations are increasingly focused on “hyperglycemia services,” where rounds are spent tracking down glucose levels and tweaking insulin doses. Outpatient electives are often cut short as residents are pulled to cover for absent colleagues or are scheduled for vacation during such rotations.

### Competing interests of service and education

Daunting responsibilities of inhopsital diabetes management for a high volume of patients epitomize the longstanding tension in medical training between service needs and education. While clinically important, the actual work is time consuming, protocol driven, documentation intensive, and, most relevant for this Perspective, it is generally unpopular with endocrinology fellows. Such dissatisfaction is readily apparent to rotating MSs and residents, and contributes to the perception of the field as an undesirable subspecialty.

### Compensation: more than salary

Considering that only 42% of endocrinologists are satisfied with their salary (13), is there an association between prospective income and intention to pursue a fellowship in endocrinology? Whereas the relationship between salary and career decisions for MSs has been established (14), the influence of remuneration on subspecialty choice is less uniform among IM residents. When stratified by gender, the selection of subspecialty positively correlated with wages in men but was inversely correlated in women (both USMGs and IMGs) (15), a finding highly relevant to the current and future endocrinology workforce. Notwithstanding methodological limitations, these studies suggest that, at least in women, factors other than salary may play a role in entering a career in endocrinology, including work–life balance and long-term patient relationships.

### Internal competition

Finally, the emergence of non-Accreditation Council for Graduate Medical Education (ACGME) 1-year training courses in diabetes or obesity medicine poses another challenge to endocrinology fellowships. While these programs serve the valuable purpose of expanding the group of clinicians managing prevalent conditions, they may also create internal competition for canonical endocrinology curricula and may exacerbate the decline in the pool of physician-scientists.

## Present and Long-term Consequences of the Reduced Supply of Endocrinologists

All domains of academic medicine and provision of care are affected by the current and projected shortage of endocrinologists.

With regard to patient care, the small workforce of adult endocrinologists, estimated to include ~6 500 physicians in 2015, is tasked with meeting the growing demand of chronic, highly prevalent diseases such as diabetes and osteoporosis. Albeit the majority of these patients will be cared for by primary care physicians, a large group of complex cases are referred to endocrinologists, especially patients with diabetes who would benefit from advanced technology options (16). The supply–demand mismatch has generated a number of detrimental consequences. From the patient’s perspective, suboptimal access, particularly in rural areas (17), reduced time spent face to face, and poor communication are the commonest complaints. On the other hand, endocrinologists feel trapped between their commitment to patient care and an expanding set of administrative tasks and volume-based reimbursement models that threaten their work–life balance, and shift the priority away from the patient–provider relationship. The net outcome is reduced patient satisfaction, and depersonalization and ultimately burnout for clinicians who will either retire or leave their group, thus amplifying the risk of burnout for providers remaining in that practice. In addition, turnover has immediate effects on costs (eg, recruitment and onboarding) and long-term implications on the stability of an organization. This scenario is even more alarming when considering that the reported rate of burnout is higher for female endocrinologists (18), who will soon represent the majority of our workforce (6).

### Who is going to mentor the new generation of clinical educators?

Even in university-based practices, increasing clinical responsibilities compete and often take priority over teaching activities, which is one of the defining pillars of academic medicine. For instance, at a time of unprecedented advances in diabetes technology, only a minority of polled fellows reported sufficient familiarity with the use and interpretation of data from insulin pumps and continuous glucose monitors (19). This gap was associated with the lack of a formal curriculum at many institutions, and suggests deficiency in teaching time and/or competency by fellowship preceptors. As discussed above, a shift in focus from education to service during fellowship training could transform many academic endocrinology divisions into “teaching private practices.” In this view, passing on the enthusiasm for teaching to the next group of endocrinology educators is fundamental for the livelihood of the entire field.

Lastly, the decreased interest in adult endocrinology fellowships has been accompanied by a dramatic drop in applications for 3-year, research-oriented positions (as noted earlier, from ~23% in 2015 to 15% in 2019). The unpredictable and increasingly competitive funding environment is a major deterrent to become a physician-scientist. When adjusted for inflation, National Institutes of Health appropriation has declined by 23% between 2003 and 2013 (20) with the mean age of first time RO1 awardees rising to 45 for MDs. Also, the number of career development (K) awards by National Institute of Diabetes and Digestive and Kidney Diseases has fallen from ~ 530 in 2009 to ~460 in 2018 (see Fig. 15B in (21)), which is worrisome because the K series represents the main vehicle to independence for early investigators including physician-scientists. Finally, time dedicated to research is often associated with a fiscal penalty for academic investigators (22), due to the lower pay scale of National Institutes of Health grants and philanthropic awards. Albeit the reduction of the stock of physician-scientists applies to many other IM subspecialties (9), it specifically affects the intellectual heritage of endocrinology as a discipline centered on innovation.

## A Multipronged Intervention Strategy

How do we make endocrinology appealing for all our trainees and promote the growth of a diverse and competitive workforce? A concerted set of changes at the policy, system, and institutional level will be required to invert the trend of fellowship applications and temper the risk of attrition for practicing endocrinologists. We will discuss a few key approaches below, understanding that many others are conceivable.

### Policy efforts

With >50% of graduating MSs reporting debt greater than $150 000 (23), measures for loan forgiveness beyond those currently available (eg, Public Service Loan Forgiveness) would be justified for IM subspecialties with lower income. Given the marked disparity in access to endocrinological care in rural communities (17), one could consider more favorable options of loan repayment for fellows who elected to practice in underserved areas after graduation. A similar program has been successfully employed as J-1 visa waiver to enhance provision of clinical services in Health Professional Shortage Areas. Such an approach could also influence workforce retention as higher debt is associated with increased rate of burnout (24).

Following the same rationale, appropriation of bridge funds for a pool of endocrinology fellows toward their first award after graduation would entice a new cadre of research-oriented fellows. This framework would hinge on the close collaboration between rigorously selected candidates and established investigators committed to mentor budding physician-scientists through the 3-year fellowship and beyond, to maximize the chance of achieving research independence.

Since these solutions do not apply only to endocrinology fellows, a joined effort from all IM subspecialties would heighten the visibility of the workforce shortage to the public and lawmakers.

### System-based solutions

The widening supply–demand mismatch begs the question of how, collectively as a consultative specialty, we can implement clinical care processes that are effective, sustainable, and aligned with professional and personal goals of our workforce (including in-training endocrinologists). A model of shared care increasingly embraced in academic centers leverages on the expertise of all team members and, equally important, mitigates physician isolation that is often a conduit to burnout. Organizations that incorporate autonomy and flexibility as important factors in workflow enjoy a high clinician retention rate. Likewise, trainees who have protected time to foster their professional goals will be more likely to achieve job satisfaction and serve as ambassadors of the field with rotating MSs and IM residents.

The progressive introduction of value-based models of care may positively impact wages for nonprocedural subspecialties and emphasize recognition for the role of the endocrinologist, thus enhancing both the appeal of our field among trainees and workforce job satisfaction.

### Committed mentors

As noted earlier in this Perspective, a more robust exposure to endocrinology during medical school and IM residency is the gateway to trainee engagement, which on the receiving hand has to be matched by a keen interest in teaching and mentoring.

In this context, one cannot overestimate the importance of the interaction of MSs and IM residents with enthusiastic endocrinology mentors—both fellows and faculty—as the lens through which younger peers see our discipline. MSs who identified role models and were favorably impressed by their educational experience in IM were also more likely to pursue a career in IM or any of its subspecialties (25). Introducing trainees to the fascinating and multidimensional aspects of endocrinology can generate a positive engagement ripple effect and a balanced perspective of the “pluses and minuses” of the field. Thus, increased emphasis on effective mentorship is a prerequisite to attract trainees into endocrinology, and to foster their career development and retention in academic medicine (26), especially at a time when burnout is affecting half of the endocrinology community (18). Because of the substantial faculty effort required by these activities, any Division of Endocrinology (and Department of Medicine) must recognize the value of this time investment in a measurable fashion.

The support of scholarly goals of students and fellows—in any dimension of academic medicine—exerts a multiplier effect. As an example, a principal investigator of a laboratory will often influence generations of other physician-scientists. More broadly, mentorship is essential to identify a path to job satisfaction, starting during residency and fellowship, and continuing through all phases of professional development. Although career priorities are likely to change over time, spending at least 20% on activities considered meaningful is associated with a much lower risk of burnout (27). The poorer level of satisfaction with mentoring and career advancement reported by females, when compared with males (7), is a gap that requires special attention.

In conclusion, the inadequate growth of the endocrinology workforce supply will have long-term ramifications on all aspects of academic medicine and clinical care. Bold measures coupled with a renewed focus on values that influence job satisfaction are required to attract top talent in endocrinology fellowships, ensure retention, and revitalize our community.

## Abbreviations

IM: internal medicine
IMG: International Medical Graduate
MS: medical student
USMG: US Medical Graduate

## Additional Information

***Disclosure Summary:*** The authors have nothing to disclose.

***Data Availability:*** All data generated or analyzed during this study are included in this published article or in the data repositories listed in References.
